# Analysing the pedigree to identify undesirable losses of genetic diversity and to prioritize management decisions in captive breeding: a case study

**DOI:** 10.1038/s41437-024-00723-z

**Published:** 2024-09-17

**Authors:** Eulalia Moreno, Isabel Cervantes, Juan Pablo Gutiérrez, Iván Fernández, Félix Goyache

**Affiliations:** 1 Departamento de Ecología Funcional y Evolutiva, Estación Experimental de Zonas Áridas (Consejo Superior de Investigaciones Científicas), Carretera de Sacramento s/n, La Cañada de San Urbano, Almería, E- 04120 Spain; 2 Departamento de Producción Animal, Universidad Complutense de Madrid, Avda. Puerta de Hierro s/n, Madrid, E-28040 Spain; 3 SERIDA-Deva, Camino de Rioseco 1225, E-33394 Gijón (Asturias), Spain

**Keywords:** Evolutionary genetics, Animal breeding

## Abstract

When prevention of species extinction is the priority, captive breeding is a key component in conservation programmes, allowing the recording of pedigree information in studbooks. The genealogical information registered in Cuvier’s gazelle studbook between 1975 and 2023 was analysed to (a) assess if the implemented mating policy was successful in preserving the genetic background of the founders (1 male:3 females) in the present population, and b) improve future management and breeding decisions. Although the maternal contribution of one founder female was lost and the mean inbreeding of the total live population was high (0.305 ± 0.095), the breeding policy applied produced better results than expected from a population starting from four founders. It was successful in keeping the individual increase in inbreeding low (0.047 ± 0.021), and, notably, the inbreeding tended to decrease during the last three decades of the breeding programme, ensuring the viability of this highly inbred population. Historical dissemination of individuals among the zoos of Europe and North America caused population structuring and genetic differentiation of the live North American population. However, it did not risk the viability of the captive population. The average relatedness coefficients allowed the identification of individuals with underrepresented genotypes, which is relevant to plan future mating guidelines to keep the founders’ representation balanced in the next generations. This study highlights the importance of keeping long-term pedigree information to monitor changes in the genetic diversity of captive populations, which is crucial to implement optimal mating decisions and assuring their long-term viability within an ex situ conservation programme.

## Introduction

For the last 40–50 years, where the focus of conservation is biodiversity, captive breeding has been a widespread maintenance technique to face the challenge of preserving many threatened species from extinction. The International Union for the Conservation of Nature has recognized the value of breeding threatened species in captivity until their reintroduction to the wild is possible (IUCN 2019). Captive breeding is mainly focused on the preservation of genetic variability to avoid the negative impacts of inbreeding. In this sense, captive breeding causes a population bottleneck due to either management practices or unexpected reproduction failure for some of the founders (Royo et al. 2007; Álvarez et al. 2008). Population bottlenecks increase inbreeding, and can lead to decreased heterozygosity, increased genetic load, and increased expression of deleterious alleles (Briskie and Mackintosh 2004). In fact, maintenance of genetic diversity is of great importance because allelic diversity can be lost at an accelerated rate when there are small, closed, and selected populations (Sheikhlou and Abbasi 2016). Therefore, avoidance of inbreeding in captive management programmes is necessary to maintain genetic variation for long-term population viability (Ballou and Lacy 1995; Ivy et al. 2009).

Apart from their major contribution to the preservation of biodiversity by educating and informing the public, the role of zoological institutions is significant in captive breeding (Conde et al. 2011) as they can function as genetic reservoirs from which wild populations can be secured if declining (through reinforcement projects) or restored if extinct (through reintroduction projects). Breeders and zoos participating in captive breeding usually try to overcome the problem of inbreeding by starting programmes with bigger possible founder populations to ensure the population’s viability (Ballou et al. 2010). However, in cases of endangered species, with extremely small remaining populations in the wild, only few individuals are available (Bock et al. 2014; Gooley et al. 2020), and conservation of genetic variability to keep the population viable is then a challenging task (Cetkovská et al. 2024).

Captive breeding programmes use studbooks to record pedigree information (Pelletier et al. 2009), which is widely recognized as one of the most important tools for both analysing the genetic relationships between individuals and making sound management decisions (Galla et al. 2022). In addition, the information in studbooks allows us to characterize the genetic diversity and gene flow of populations through pedigree analyses (Lozada et al. 2023). However, analyses based on pedigree data depend mostly on the completeness and accuracy of the recorded data, which are crucial for obtaining reliable results (Siderits et al. 2013). Although the increasing availability of molecular technology helps us to clarify individual relatedness in pedigrees (Oliehoek and Bijma 2009; Galla et al. 2020), complete and accurate pedigrees have been shown to explain more the variation in inbreeding than microsatellites (Nietlisbach et al. 2017) and to provide similar estimates to thousands of SNPs (Galla et al. 2020). Therefore, the potential benefit of pedigree analysis for guiding genetic management and improving long-term genetic viability should not be neglected, as zoos use pedigree information in a well-supported paradigm of measuring and managing putative genome-wide neutrality (Galla et al. 2020). While molecular techniques can help overcome pedigree challenges such as missing data, robust pedigrees allow for more efficient and economical research work (e.g. avoiding massive genotyping; Perdomo-González et al. 2022), making them still a relevant tool in conservation genetics, which will be even more powerful if combined with molecular data (Ayala-Burbano et al. 2020).

Here we analyse the pedigree information included in the international studbook of Cuvier’s gazelle (*Gazella cuvieri*, Ogilby 1841), an ungulate species of the Family Bovidae listed in the International Union for Conservation of Nature Red List as “vulnerable” (IUCN 2019). Its captive breeding programme was established at “La Hoya” Experimental Field Station in 1975 (EEZA-CSIC; Almeria, Spain; Cano 1988), when four individuals (2 males and 2 females) arrived from a captive population in Morocco (Escos 1992). In September 1986, a new dam bloodline came into the programme (Abáigar and Cano 2005), which is since 2006 under the auspices of the European Association of Zoos and Aquaria and the World Association of Zoos and Aquaria. The species is being intensively managed on the basis of studbook information. Pairing strategies in Cuvier’s gazelle captive breeding programme have been set up primarily using the SPARKS software programme (ISIS 2004) and then using PMx (Ballou et al. 2011) following the criteria of minimizing the coancestry between mating individuals.

With the general aim of helping and improving the future management of its whole captive population as well as the breeding decisions, our specific objectives were the following:

1. To describe the historical progress of the species’ captive population and some demographic parameters since it started in 1975. Demographically, the first aim of captive breeding programmes is to rapidly increase the population size to avoid extinction and maintain reliable reproduction.
2. To evaluate how the breeding programme implemented in 1975 for the captive population of Cuvier’s gazelle fitted with the goal of maintenance of the genetic diversity in the species across years and generations. Genetic diversity can eventually decline and inbreeding increase in captivity owing to small effective population sizes and adaptation to captivity due to the new selection pressures associated with captive breeding practices (absence of predators, provisioning of food, and medical treatment).
3. To assess the genetic representation of the founders of the captive breeding programme in the live population of Cuvier’s gazelle. Analysis based on pedigree can be employed to assess how large shares of the present population genome can be traced back to each individual founder in the wild-caught stock. This will inform on a possible imbalance of the different founder lines in the present population, therefore, allowing the identification of live individuals that must be used preferentially when making mating decisions.
4. To assess if the dissemination of Cuvier’s gazelle across the world could have caused population structuring affecting its genetic diversity. Knowledge of population structure combined with information of genetic changes in the population can be used to implement future management strategies to guide the preservation of the extant genetic stock in the programme.

## Materials and methods

### Study species, data set, and reference populations

Cuvier’s gazelle is a threatened ungulate listed in the International IUCN (2019) Red List as “vulnerable”. This species declined dramatically since the 1950s of the XXth century (Beudels et al. 2005; Beudels et al. 2013), apparently due to excessive hunting, anthropogenic barriers, and habitat degradation (IUCN 2018). Some details of its biology are given in appendix S1 of Supplementary Material, and extensive information can be found in IUCN 2018.

**Fig. 1:**
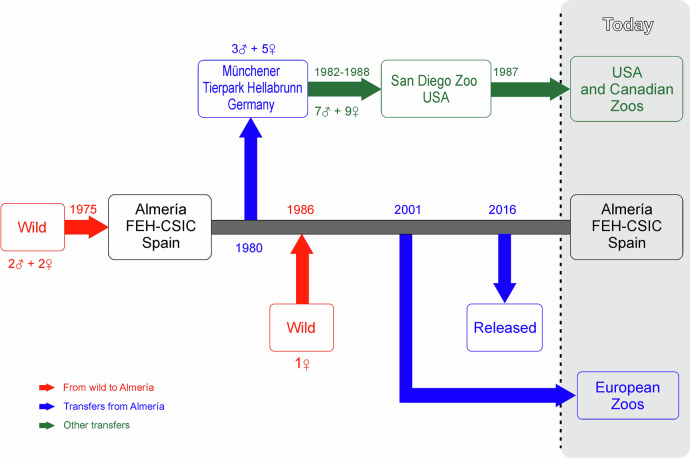
History line of the Cuvier’s gazelle captive breeding programme (1975–2023). Incomes (number of individuals) from the wild (red) to Finca Experimental La Hoya (Almeria) and main transfers from it are shown: in blue if directly transferred from Almeria; in green if indirectly, using Münchener Tierpark Hellabrunn as an intermediate zoo. FEH-CSIC Finca Experimental La Hoya.

The history of Cuvier’s captive-bred gazelle programme is shown in Fig. 1. Four individuals—two males and two females—came from a captive population in the Oued Draa Valley (Tan-Tan, Morocco; Escos 1992) to “La Hoya” Experimental Field Station in 1975. Of these two males, only one was used as a breeder (Escos 1992). In September 1986, a new dam bloodline came into the programme through a female imported from the Medium Atlas Mountains in Morocco to a private property in Almeria (Escos 1992; Abáigar and Cano 2005). In October 1980, 8 individuals (3 males and 5 females) were transferred from “La Hoya” to Münchener Tierpark Hellabrunn (Moreno and Espeso 2008). Between 1982 and 1988, 16 Cuvier’s gazelles (7 males and 9 females) were transferred from Münchener Tierpark Hellabrunn to San Diego zoo (Moreno 2023), from where individuals of Cuvier’s gazelle spread throughout several zoos in the United States and Canada. Hence, along the history of the Cuvier’s gazelle captive breeding programme, “La Hoya” has been the unique source of genes of the captive population and the spreading of individuals to different zoological institutions either directly (from “La Hoya” to European zoos) or indirectly (from “Lo Hoya” to Münchener Tierpark Hellabrunn and from here to North American zoos). Due to unknown reasons, likely logistic, between 1990 and 2001 there was no transfer of animals from “La Hoya” elsewhere (Moreno 2023).

The genealogical data of Cuvier’s gazelle captive population have been recorded in its studbook for 48 years (1975–2023), which provides a valuable opportunity to perform analyses based on its pedigree. The data set for this study is that included in Cuvier’s gazelle International Studbook updated at 31 May 2023 (Moreno 2023). The variables in the studbooks are as follows: studbook number (unique number identifying the individual within the population), sex type, birth date, sire, dam, birth location, event type (variable with 4 categories: birth, death, transfer from one place to another, and loss to follow-up), and date and location of occurrence of each event. From our raw data set (1884 records), we removed records with ambiguous parental identification, birth date, sex, or location (for any event) of the individual. After editing, the whole pedigree included 1777 records (877 males and 900 females). Of them, up to 1772 were born in captivity (876 males and 896 females) from 1262 different litters (757 singleton, 500 twins, and 5 triplets). Five hundred and seventy-two individuals (158 males and 414 females) had offspring as per the data.

One hundred and fifty-three individuals were live at the moment of analysis (Table 1). This **total live** population was subdivided, according to geography, into three different populations: 1. **Almeria**, 2. **Europe**, and 3. **North America**. We also included in the dataset 37 individuals released in a Tunisian national park in 2016 within the framework of a reintroduction project (see Moreno et al. 2020 for a detailed description of the project and its main demographic results). After reintroduction, neither these individuals (hereafter referred as **released**) nor their descendants were available for further management measures to be implemented in the captive population of Cuvier’s gazelle but rather were available to reinforce reintroduction when needed (Alvarez-Estapé et al. 2022). These five populations (total live, Almeria, Europe, North America, and released) were considered reference populations for the genetic analyses. They are expected to inform on (i) the present genetic scenario of the whole captive population of the species, (ii) the possible structuring of the population and its impact on genetic diversity, and (iii) the representativeness of the individuals used in the reintroduction project of the genetic background of the species. For descriptive purposes, most genetic parameters will be given for the whole pedigree as well.

**Table 1:** Census sizes and females and males of the live captive Cuvier’s gazelles at the time of the analysis (31 May 2023).

	Females	Males	Total
Total live	88	65	153
Almeria	45	35	80
Europe	17	18	35
North America	26	12	38
Released	31	6	37

The number of individuals in the total live population, as well as those included in every subpopulation considered in the analysis, is shown.

### Parameters computed

Several demographical and genetic parameters were computed using the program ENDOG v.4.8 (Gutiérrez and Goyache 2005). Where necessary, a detailed description of the computation carried out is given in appendix S2 of Supplementary Materials. The list of parameters computed is the following:

- Generation intervals, computed as the average age of parents at the birth of their progeny kept for reproduction using the birth dates of the registered animals together with those of their sires and dams. The four pathways (sire–son, sire–daughter, dam–son, and dam–daughter) were considered.
- The number of equivalent complete generations traced (*t*; Maignel et al. 1996).
- The inbreeding coefficient (*F*), defined as the probability that two alleles at a randomly chosen locus are identical by descent (Malècot 1948).
- The average relatedness coefficient (AR), defined as the probability that an allele randomly chosen from the whole pedigree belongs to a given animal (Goyache et al. 2003). It was computed for each individual included in the studbook analysed. Note that the AR coefficient of a founder means its genetic contribution to the population. For each reference population, these coefficients can be summed up for the founders to ascertain their relative contributions to the studied population.
- The probability of gene origin was characterized by computing the following parameters: i) effective number of founders (*f*_*e*_), which is the reciprocal of the probability that two alleles drawn at random in the studied population originate from the same founder (James 1972), computed from the genetic contribution of founders to the descendant gene pool of the population (Lacy 1989); ii) effective number of ancestors (*f*_*a*_), defined as the minimum number of ancestors, not necessarily founders, explaining the complete genetic diversity of a population (Boichard et al. 1997) [Parameter *f*_*a*_ does not fully account for gene loss by drift from the ancestors to a reference population, but complements the information offered by *f*_*e*_ accounting for the losses of genetic variability produced by the unbalanced use of reproductive individuals producing bottlenecks (Boichard et al. 1997; Gutiérrez et al. 2005).]; and iii) the founder genome equivalents (*f*_*g*_; Ballou and Lacy 1995), defined as the theoretically expected number of founders that would be required to provide the genetic diversity in the present population if the founders were equally represented and had lost no alleles, obtained by the inverse of twice the average coancestry of the individuals within each reference population (Caballero and Toro 2000). Finally, the effective number of non-founders (*nf*_*e*_) was computed following Caballero and Toro (2000).
- Effective population size (*N*_*e*_) was estimated using the individual increase in inbreeding, Δ*F*_i_ (Gutiérrez et al. 2009; ${N}_{e}{F}_{i}$), and coancestry, Δ*C*_ij_ (Cervantes et al. 2011; ${N}_{e}{C}_{{ij}}$).
- Following Caballero and Toro (2000, 2002), *F*_*IS*_ and *F*_*ST*_ statistics were computed from coancestry.
- Contributions of geographical populations to total gene diversity were assessed following Caballero and Toro (2002). Note that, in this approach, positive values for the contributing subpopulations indicate that the remaining dataset increases the overall diversity when the subpopulation is removed. Consequently, that subpopulation would not contribute significantly to the overall diversity and would not be preferred for conservation.
- The contribution of the three founder dam lines registered in the studbook to the five reference populations will be assessed according to Álvarez et al. (2012).

## Results

### Demographic parameters

**Fig. 2:**
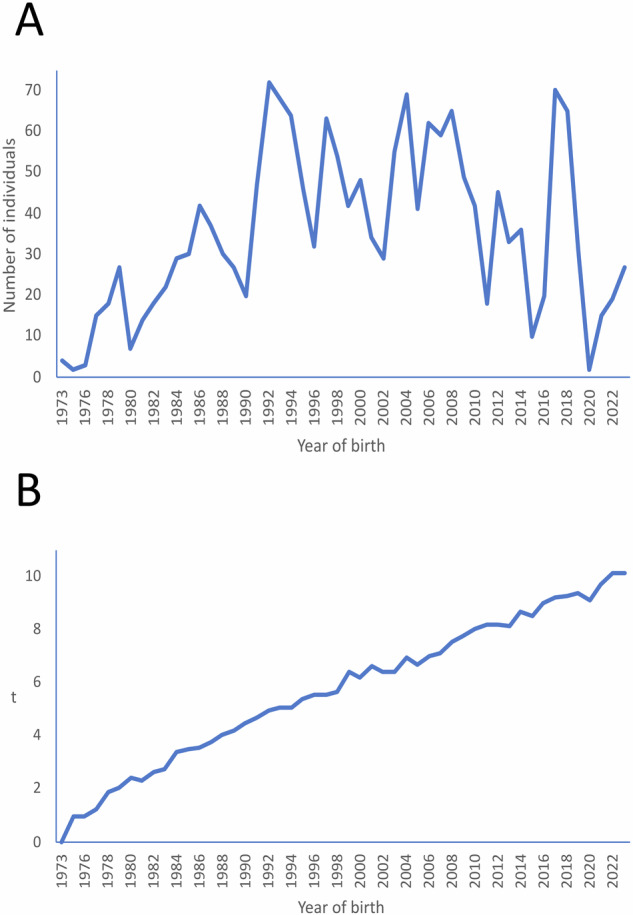
Demographic trends of the Cuvier’s gazelle captive breeding programme. It starts in 1973, as, according to the studbook, this is the date when founders were born. Number of entries (Plot **A**) and mean equivalents to complete generations (*t*; Plot **B**) by years of birth of the individuals registered in the studbook of the Cuvier’s gazelle.

The number of individuals yearly registered in the studbook steadily increased since the foundation of the breeding programme to reach a maximum of 72 individuals in 1992 (Fig. 2A). From 1992 to 2018, registrations kept at a good pace (about 47 individuals a year on average), tending to decrease from 2019 to present (about 19 individuals registered a year on average). The pedigree depth of the individuals registered steadily increased with year of birth (Fig. 2B) to reach a maximum of 10.1 equivalents to complete the generations in 2022 and 2023. From 1992 to 1994, the mean *t* of the individuals registered was about 5, and from 2019 thereafter, *t* was higher than 9. The average generation interval computed was 4.9 (±0.07) years. The sire pathways were always higher than 5 years. The dam pathways were always shorter than the sire pathways by about 0.3 to 0.6 years (Table 2).

**Table 2:** Mean generation intervals (±standard error), in years, for the four pathways of parent-offspring computed using the whole pedigree of *Gazella cuvieri*.

Pathway	N	Years
Sire–son	158	5.1 (±0.19)
Sire–daughter	421	5.2 (±0.13)
Dam–son	158	4.8 (±0.18)
Dam–daughter	421	4.6 (±0.12)
Average	1158	4.9 (±0.07)

*N* is the number of parent-offspring couples used for the calculations.

### Parameters characterizing genetic diversity

**Fig. 3:**
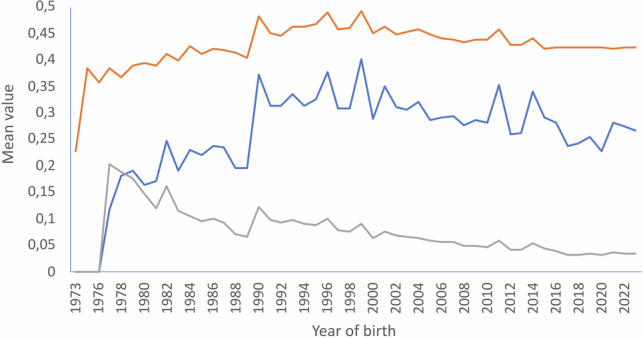
Temporal variation of three genealogical parameters in the studbook of Cuvier’s gazelle. The orange, blue, and grey lines illustrate the mean values of average relatedness (AR), individual coefficient of inbreeding (*F*_i_), and individual increase in inbreeding (Δ*F*_i_), respectively.

Figure 3 illustrates the variation of average relatedness (AR; in orange), inbreeding (*F*_i_; in blue), and individual increase in inbreeding (Δ*F*_i_; in grey) by year of birth of the individuals registered in the *Gazella cuvieri* studbook. Mean individual inbreeding increased after the start of the breeding programme and reached a mean value of *F*_*i*_ = 0.371 in 1990. Then, the mean *F*_*i*_ kept above 0.3 until 1999, in which a peak value of *F*_*i*_ = 0.401 was reached. Thereafter, the mean inbreeding of the individuals born tended to be below 0.3. AR followed a similar pattern to *F*_*i*_, reaching the higher mean values in 1990 and 1999 (0.485 and 0.494, respectively). Notably, regardless of the constant accumulation of pedigree knowledge (Fig. 2B), which is expected to cause the accumulation of inbreeding and coancestry in a small and closed population, the individual increase in inbreeding showed a steady declining pattern since 1990, with mean values of about 3% from 2016 to present (Fig. 3).

Table 3 gives the parameters characterizing genetic diversity for the whole pedigree of Cuvier’s gazelle, as well as for the five reference populations defined: total live, Almeria, Europe, North America, and released populations. The average number of equivalents to complete generations in the total live population was *t* = 9.0 ± 0.9. This parameter had a higher mean value in the Almeria and the released (9.5 and 9.4, respectively) populations, and had a lower value in the North American (8.1 ± 0.7) population.

**Table 3:** Parameters characterizing genetic variability and the probability of gene origin of the animals registered in the studbook of *Gazella cuvieri*.

	Whole pedigree	Total live	Almeria	Europe	North America	Released
Total number of animals in the population	1777	153	80	35	38	37
Equivalents to complete generations (*t*)	6.2 ± 2.1	9.0 ± 0.9	9.5 ± 0.8	9.0 ± 0.6	8.1 ± 0.7	9.4 ± 0.3
Deficit of heterozygotes due to populations subdivision *(F*_*IS*_*)*	0.081	0.080	−0.034	−0.041	−0.049	−0.074
Inbreeding coefficient (*F*)	0.283 ± 0.128	0.305 ± 0.095	0.261 ± 0.050	0.253 ± 0.036	0.446 ± 0.056	0.239 ± 0.017
Average Relatedness (AR)	0.439 ± 0.051	0.443 ± 0.035	0.423 ± 0.002	0.423 ± 0.003	0.506 ± 0.007	0.424 ± 0.002
Individual increase in inbreeding (Δ*F*_*i*_)	0.074 ± 0.054	0.047 ± 0.021	0.035 ± 0.008	0.036 ± 0.007	0.080 ± 0.009	0.032 ± 0.004
${N}_{e}{F}_{i}$	6.8 ± 1.7	10.7 ± 1.5	14.2 ± 0.8	13.9 ± 0.7	6.2 ± 0.3	15.6 ± 0.4
${N}_{e}{C}_{{ij}}$	10.3 ± 0.7	15.4 ± 0.6	14.0 ± 0.3	14.3 ± 0.3	6.5 ± 0.2	14.3 ± 0.3
*mN*_*e*_	4.0	3.7	2.8	3.2	1.0	1.2

Parameters are given for the whole pedigree, and for the five reference populations: fitted Total live (all individuals live as at 31 May 2023), Almeria, Europe, North America (individuals live in Almeria, Europe, and North America as at 31 May 2023), and in the Released population.

Parameter *F*_*IS*_ (deficit of heterozygotes due to the population’s subdivision) took positive and high values in both the whole pedigree (0.081) and the total live population (0.080) because of unavoidable matings between relatives. However, parameter *F*_*IS*_ took negative values in all other reference populations: Almeria, Europe, North America, and, particularly, released (−0.074).

Inbreeding coefficient (*F*_*i*_), AR, and Δ*F*_*i*_ values computed for the total live population were 0.305 ± 0.095, 0.443 ± 0.035, and 0.047 ± 0.021, respectively (Table 3). Almeria, Europe, and released populations had lower values for these three parameters, whereas the North American population had substantially higher values (*F* = 0.446 ± 0.056; AR = 0.506 ± 0.007; and Δ*F*_*i*_ = 0.080 ± 0.009).

The estimates of *N*_*e*_ given in Table 3 varied with both the methodology used and the population assessed. Estimates of ${N}_{e}{F}_{i}$ and ${N}_{e}{C}_{{ij}}$ for the total live population were 10.7 ± 1.5 and 15.4 ± 0.6, respectively. However, the Almeria and released populations had higher *N*_*e*_ values when the inbreeding-based method was used, which is reflective of the low values assessed for Δ*F*_*i*_ in such populations. The North American population showed lower *N*_*e*_ values, with ${N}_{e}{F}_{i}$ (6.2 ± 0.3) exceeding ${N}_{e}{C}_{{ij}}$ (6.5 ± 0.2) because of the different breeding history of that geographical population.

Parameter *mN*_*e*_ showed a value of 3.7 in the total live population. Although in the Almeria and Europe populations maternal effective size had lower values (2.8 and 3.2, respectively), they were substantially higher than in the released (1.2) and North American populations (1.0). These low *mN*_*e*_ values were due to a very unbalanced representation of the three registered dam lines in the released population and the absence of one of them (female 191) in the North American one.

### Probabilities of gene origin

Four individuals acted as founders in the captive breeding programme of Cuvier’s gazelle: male studbook number 1, which arrived at “La Hoya” in 1975; female studbook numbers 2 and 3, which arrived at “La Hoya” in 1975 at the same time as that of male number 1; and female studbook number 191, whose bloodline came into the programme in 1986 (see Fig. 1 and Table 4). They were also identified as ancestors (breeding individuals, founder or not, summarizing the genetic variability of the reference population), therefore suggesting that the breeding policy applied avoided losses in genetic diversity caused by the occurrence of bottlenecks due to an overuse of some individuals for reproduction. In such an unusual scenario, the genetic contributions to the population as either founders or ancestors (as well as *f*_*e*_ and *f*_*a*_) coincided (Table 4). Whatever the reference population considered, the lower genetic contribution of a founder (0.156 or lower) corresponded to female 191, which, in addition, has never been present in North America. The male founder had always the higher genetic contributions to the populations fitted, exceeding 50% (0.510) in the North American population. The contributions of the four founders to two of the geographical populations (Almeria and Europe) and to the released population were highly consistent. The male founder explained somewhat more than a third of the genetic variability (37.5%), with female number 3 contributing the most among the dams (about 27%), followed by female number 2 (about 19%) and female number 191 (roughly 15.5%). However, this scenario was not the same in the North American population, in which the genetic background of female 191 was not represented.

**Table 4:** Description (studbook number, sex, year of birth, and number of offspring) and their genetic contributions to the diversity of the individuals acting as founders and ancestors in the whole pedigree registered in the Cuvier’s gazelle studbook (whole), and the five reference populations studied (total live, Almeria, Europe, North America, and released into the wild).

				Explained variability in each population					
Studbook number of the individual	Sex	Year of birth	Offspring	Whole	Total live	Almeria	Europe	North America	Released
1	male	1973	20	0.429	0.408	0.374	0.376	0.510	0.375
2	female	1973	8	0.207	0.199	0.189	0.190	0.228	0.190
3	female	1973	13	0.277	0.276	0.281	0.278	0.262	0.279
191	female	1981	2	0.087	0.117	0.156	0.155		0.156
Probability of gene origin									
Total number of founders				4	4	4	4	3	4
Effective number of founders (*f*_*e*_)				3.2	3.4	3.6	3.6	2.6	3.6
Effective number of ancestors (*f*_*a*_)				3.2	3.4	3.6	3.6	2.6	3.6
Founder genomes equivalents (*f*_*g*_)				2.3	2.0	1.8	1.8	1.1	1.7
Effective number of non-founders (*nf*_*e*_)				8.2	4.9	3.6	3.6	1.9	3.2

Parameters characterizing the probability of gene origin of the animals registered in the studbook of *Gazella cuvieri* (*f*_*e*_, *f*_*a*_*, f*_*g*_, and *nf*_*e*_) are given. Note that parameter *f*_*a*_ coincides with parameter *f*_*e*_.

The effective number of founders (*f*_*e*_) was 3.4 in the total live population and 3.6 in the released, Almeria, or Europe populations. However, the North American population had a lower *f*_*e*_ value (2.6). The founder genome equivalents (*f*_*g*_), characterizing losses of genetic diversity caused by all drift sources, was 2.0 for the total live population, meaning that about half of the founders’ genetic background is not in the present population (ratio *f*_*g*_/*f*_*e*_ = 0.59). Founder genome equivalent (*f*_*g*_) reached slightly lower values for the released and both the Almeria and the Europe populations (from 1.7 to 1.8). However, in the North American population parameter *f*_*g*_ took a value of 1.1, suggesting that the genetic background of roughly two out of the three founders of that population was lost (ratio *f*_*g*_/*f*_*e*_ = 0.42). The effective number of non-founders was more than two-fold higher than *f*_*e*_ in the whole pedigree population and 1.4-fold higher in the total live population, suggesting that most genetic variability of the species is gathered by the individuals born after the start of the breeding programme. However, this general trend could not be assessed at the geographical populations’ level: parameter *nf*_*e*_ equalled *f*_*e*_ in the Almeria and Europe populations and was lower than *f*_*e*_ in the North American and released populations. This means that, although mating policy could have avoided a population bottleneck at the species level after the start of the captive breeding programme, the geographical populations created from that of Almeria not always included a balanced representation of all founders, leading to the occurrence of a bottleneck-like event at least in the North American and released populations.

### Differentiation between populations

The differentiation between the North American and other populations fitted was high, varying from *F*_*ST*_ = 0.124 with Almeria to *F*_*ST*_ = 0.149 with the released population (Table 5). However, the differentiation between the Almeria and both the Europe (*F*_*ST*_ = 0.017) and released (*F*_*ST*_ = 0.021) populations was very low. The coancestry between the North American population and both the Almeria and Europe populations was relatively low (0.166), whereas the pair Almeria-Europe showed higher genetic identity (0.254; Table 5). The individuals belonging to the North American population showed a high genetic identity (mean *f* = 0.472).

**Table 5:** Number of samples (*N*), within-populations mean coancestry (*f*), and contributions (in percentage) to within-population, between-population and total contributions to *N*_*ei*_*’s* gene diversity of each of the geographical populations of *Gazella cuvieri* analysed.

		Contributions to gene diversity			Between-population F_ST_’s and coancestry			
Populations	*f*^a^	gGD_W_	gGD_B_^b^	gGD_T_	1	2	3	4
1. Almeria	0.285	0.328	−0.447	−0.119		0.254	0.166	0.252
2. Europe	0.282	0.131	−0.175	−0.044	0.017		0.166	0.263
3. North America	0.472	0.675	−0.507	0.168	0.124	0.145		0.166
4. Released	0.292	0.166	−0.212	−0.046	0.021	0.017	0.149	

^a^includes self-coancestries

^b^corresponding to N_ei_’s minimum distance.

Furthermore, between-population *F*_*ST*_*’s* (below diagonal) and coancestry (above diagonal) are given.

*gGD*_*W*_ contribution to within-population gene diversity, *gGD*_*B*_ contribution to between-population gene diversity, *gGD*_*T*_ total contribution to gene diversity.

The importance of each geographical population for the maintenance of the genetic diversity of Cuvier’s gazelle was also assessed in terms of gene diversity (Caballero and Toro 2002). The most favourable total contribution to diversity was assessed for the Almeria population (−0.119). However, the Europe and released populations had also favourable contributions to diversity (−0.044 and −0.046, respectively), suggesting that these geographical populations may act as partial genetic reservoirs for the species. The contribution of the North American population was unfavourable (0.168), suggesting that its genetic background is well represented in the other populations defined. In any case, contributions to diversity are mainly due to the between-population component (gGD_B_; always having negative values), while the within-population component (gGD_W_) was always unfavourable. This would suggest that the individuals forming the four populations fitted have a significant genetic identity, particularly in the case of the North American population (0.675).

## Discussion

### Demography

Captive breeding is a key component in many threatened species conservation programmes, its priority being preventing species extinction (Swinnerton et al. 2004) and then to begin managing the populations towards growth and self-sustainability (Millar et al. 1997). Maintaining genetic diversity is important, but also an increase in population size as rapid as possible. These two goals have to be compatible, meaning that increasing the population size should not result in the loss of genetic diversity and retaining genetic diversity should not influence the population growth rate. In our study case, the extremely low number of individuals used as founders (1 male, 3 females) made mating between close relatives unavoidable, mainly at the beginning of the breeding programme. But, contrary to our expectation, the number of individuals born per year increased gradually up to 72 in 1992 (Fig. 2A), which suggests that the mating strategy used was successful in avoiding inbreeding depression, at least in terms of female productivity [see Moreno and Espeso (2008) for data on population growth between 1975 and 2007].

Estimates of inbreeding and relatedness are highly dependent on both the quality and the depth of the available genealogies. However, there exists evidence suggesting that most information on relatedness is captured when genealogies of the most recent three to five generations have good quality, with estimates of inbreeding changing little as deeper links are added (Balloux et al. 2004). The pedigree analysed here ensures that all individuals can be traced back to the founders of the captive population. Therefore, the results presented are not estimates suggesting population trends, but an accurate description of the demographic and genetic scenarios of the species.

The mean generation interval computed for the Cuvier’s gazelle captive population (4.9 years) can be regarded as high considering that females are sexually mature at the age of 8–9 months and males at 12–13 (Moreno and Espeso 2008), and, compared with other domestic species, reaching sexual maturity at a similar age (see Goyache et al. 2003, for a review). Lengthening of generation intervals is a usual strategy in the management of captive small populations to avoid the accumulation of inbreeding. Interestingly, in the sire–offspring pathways, the generation interval was higher, perhaps because of the slow rate of replacement of males in the breeding groups between 1975 and 2001 (Escos 1992; Moreno and Espeso 2008), which, in turn, lengthened the reproductive career of the founders and their descendants as much as possible to keep their genetic contributions in the population. The extended longevity of males (Moreno and Espeso 2008) could have played a role as well.

### Performance of the captive breeding programme

The endangered Cuvier’s gazelle captive breeding programme started in 1975 is based on four breeding founders (1 male:3 females). Under this circumstance, it was almost impossible to implement matings between unrelated individuals. This has represented a constant threat to the viability of the population due to the risk of inbreeding depression. However, the current analysis illustrates that the breeding policy implemented over 48 years has been mostly successful in preserving this genetic stock in the long term, avoiding apparently adaptation to captivity (Moreno et al. 2020). Although inbreeding (*F*) and average relatedness (AR) tended to keep high values across the years due to the matings implemented mainly during the initial period of the programme (1975–1990; Fig. 3), the individual increase in inbreeding (Δ*F*_i_) per year of birth remained low and even tended to decrease during the last three decades of the breeding programme (1992–2022; Fig. 3). This is crucial to ensure the viability of a small, highly inbred population: *N*_*e*_ tended to increase. At any given level of inbreeding, inbreeding depression would be less probable in individuals accumulating inbreeding over a larger number of generations (Van Wyk et al. 2009). Slow accumulation of inbreeding allows selection to operate and to remove the less-adapted animals. It also permits the genomic burden of putatively deleterious alleles to be purged from the population, as demonstrated by López-Cortegano et al. (2021) in captive Cuvier’s gazelle.

Furthermore, the breeding policy has been successful in avoiding the occurrence of additional bottlenecks in the population that would had occurred if the mating policy had allowed an overuse of some individuals for reproduction (Menéndez et al. 2016). Indeed, losses of genetic diversity occurred. Parameter *f*_*g*_, which summarizes all causes of losses of genetic diversity, was below 2 in all the geographical and released populations (Table 4), and the ratio *f*_*g*_*/f*_*e*_ was 0.5 or lower. Moreover, the current analyses ascertained that the maternal contribution of one of the female founders (191) was lost (she had only one daughter and no granddaughters), although their genes are still present following the male path, except for the North American population. In the same direction, parameter *f*_*a*_ (equalling *f*_*e*_) informed that no overuse of individuals for breeding occurred, and *n*_*fe*_, in most cases equalling or being always higher than *f*_*e*_, informed that the random losses of genes after the breeding programme started were minimized. In the case of Cuvier’s gazelle, the mating policy applied seems to have produced better results than expected from a population starting from only four founders. In genealogical terms, parameter F_IS_ is equivalent to the parameter alpha proposed by Caballero and Toro (2000) and informs on the avoidance (or not) of matings between relatives, thus characterizing the breeding policy. It is worth noting that the F_IS_ values computed for the geographical populations were always negative, confirming that matings are strictly planned to avoid those between the closest relatives available.

For small populations, Franklin (1980) suggested, as a rule-of-thumb, that the effective population size (*N*_*e*_) should not be <50 in the short term, and should not be <500 in the long term. The first figure (50) is intended to avoid the harmful consequences of inbreeding depression, while the second (500) is intended to retain enough quantitative genetic variation to allow future adaptive change. This 50/500 rule, although subject to criticism (Jamieson and Allendorf 2012), is used as a guiding principle to indicate when genetic concerns are likely to have an important role in the short- and long-term viability of populations. In this study, any estimate of *N*_*e*_ computed for the Cuvier’s gazelle is substantially lower than 50. However, except for the North American population, estimates of ${N}_{e}{F}_{i}$ and ${N}_{e}{C}_{{ij}}$ are roughly four- and three-fold the actual number of founders (*N* = 4), respectively, suggesting that the breeding policy applied has been successful in giving the viability of the captive population of the species a chance. López-Cortegano et al. (2021) found estimates of *N*_*e*_ similar to ours for both Cuvier’s and dama gazelles (*Nanger dama mhorr*) in captivity. In captive Cuvier’s gazelle, purging has previously been evidenced to yield a fitness trait such as juvenile survival, because it induces a substantial fitness recovery in the population (Moreno et al. 2015; López-Cortegano et al. 2021). *N*_*e*_ is a key parameter indicative of the evolutionary ability of a population. The values estimated, together with the good performance of the Cuvier’s gazelle individuals in growth and reproduction (Moreno et al. 2015), suggest that the captive population of the species can be viable in the midterm if the breeding policy does not deviate much from that implemented up to now. This also applies to the production of individuals aimed for use in the initiative of reintroduction of the species in its natural habitat. In general, the released population has genealogical parameter values suggesting that there are no genetic concerns challenging the success of such initiatives, in accordance with the results obtained by Álvarez-Estapé et al. (2022) at a molecular level in such a released population.

### Current founder representation and future management strategies

The general scenario of the captive Cuvier’s gazelles depicts a demographically fragile, highly inbred, population. The current analysis allowed ascertaining that geographical dissemination of the Cuvier’s gazelle individuals in the 1980s caused population structuring yielding a regional excess of inbreeding with regards to coancestry that, theoretically, can challenge the viability of the North American population or the flocks forming from this reference population. The main cause of this structuring was that the individuals transferred in 1980 from Almeria (La Hoya Field Station) to Munich (Münchener Tierpark Hellabrunn) did not include the descendants of one of the female founders (number 191 was included in the breeding programme in 1986). Accordingly, the stock moved from Munich to North America (San Diego Zoo) did not include dam 191 bloodline either. However, the propagation promoted since 2002 onward to disseminate Cuvier’s gazelles from Almeria to new host institutions in Europe (Fig. 1; Moreno 2023) was performed in such a way that founders’ representation in them paralleled that observed in the nucleus (source) of Almeria (Table 4). The same selection procedure was used to choose the founder stock to be released into the wild in Tunisia (Moreno et al. 2020; Table 4). This caused very low F_ST_ values in these three populations (Table 5), meaning that, at least in the case of Almeria and the current European populations, most of the genetic variability of the species is kept and shared, and it is available for later use in conservation actions (reintroduction and reinforcement projects). In other words, despite the overwhelming importance of the Almeria population, the future of the captive population of the species is not dependent on the breeding success at La Hoya Experimental Field Station (Almeria).

In this respect, our analyses confirm that the total live population is structured and that this structuring is caused by the formation of the North American population. Unlike geographical populations, the ${N}_{e}{C}_{{ij}}$ largely exceeds ${N}_{e}{F}_{i}$ in the total live population (Table 3). The ratio $\frac{{N}_{e}{C}_{{ij}}}{{N}_{e}{F}_{i}} > 1$, indicating that inbreeding substantially exceeds coancestry, has been suggested as a measure of population structuring (Cervantes et al. 2008). Furthermore, the analysis of contributions to diversity confirms that the North American population is highly differentiated from the others (with the gGD_B_ component being high and negative). However, the total contribution to diversity of this geographical population suggests that the genetic diversity of the North American Cuvier’s gazelle is well represented in the other geographical populations and, therefore, the existence of population structuring at the live population level does not risk the viability of the captive population of the species.

The current analysis confirms that losses of genetic variability have occurred, and that the implementation of measures for improving this situation is advised in this captive population. However, some of the losses (e.g., the contribution of female founder 191 on the maternal side) are unrecoverable, and the unbalanced representation of the founders in each geographical population may challenge their viability, particularly in the case of the North American population. When the genetic representation of some lines of founders outweighs that of others, an accepted strategy to preserve genetic variability in a small population is to unbalance the genetic contributions of individuals belonging to the less-represented genetic lines to equalize the genetic representation of the founders in the whole population (Ballou and Lacy 1995). In this respect, parameter AR has been proposed to monitor pedigrees (Goyache et al. 2003; Royo et al. 2007; Álvarez et al. 2008). Descendants of under-represented founders (i.e., those with relatively low AR values) are identified and optimal matings are decided according to the actual coancestry between possible couples. This way, offspring in the further few breeding seasons are unbalanced as much as possible towards those animals to maintain the initial genetic variability and to control the average AR values in the new stock. In the case of the North American population, only the addition of individuals descending from the female founder 191 would erase the genetic differences assessed with other geographical populations. In the case of the Almeria and Europe populations, breeding policy should consider the preferential use of individuals descendant from founders 2 and 191 with the lowest AR value (i.e., with the lowest genetic representation in the population) as parents (dams) of the next generations. By doing this, their genetic representation would increase tending to balance that of the female number 3 and to get closer to that of the only founder male (Table 4). The exchange of breeding individuals between populations should also be considered as an advisable strategy to improve the genetic variability in future generations.

Captive breeding programmes of endangered species frequently involve the use as founders of a low number of individuals from a genetically depleted source population where a dramatic population bottleneck occurred. It necessarily involves mating between relatives, which leads to the loss of genetic variation that is the raw material of evolutionary change. Analysis of genealogical information is much useful to monitor genetic variability in small captive populations of endangered species. The identification of founder lines with low contributions to the present population and the ascertainment of unexpected losses of genetic diversity provide insights to improve the global breeding policy and to ensure the viability of the species. In the Cuvier’s gazelle population, one of the males (studbook number 6) that arrived in Almeria in 1975 did not breed at all, which represents an undesirable loss of genes for the captive breeding programme. The maternal contribution of female number 191 is also lost in the current populations, although part of their genes are still present following the male path. Therefore, future management decisions for the whole captive population of Cuvier’s gazelle should prioritize obtaining viable offspring of underrepresented founder lines, particularly the one not represented in the North American subpopulation (female 191). It is also advised to obtain descendants from dam lines 2 and 191. Implementation of these strategies would sometimes require moving individuals between continents, but sanitary reasons made these movements impossible most times (sometimes even among countries). When planning reintroduction and reinforcement, stocks to form multi-origin cohorts has been proven to be a good strategy to ensure a balanced genetic representation of the total genetic background of the species’ captive population.

Our results illustrate the value of studbooks, long-term conservation datasets, for analysis of the genetic processes associated with captive breeding (see Domínguez et al. 2024 for similar results in captive mohor gazelles). They are the tool of choice for estimating relatedness in hundreds of conservation breeding practices managed by the worldwide zoo and aquarium community (e.g., the European Association of Zoos and Aquariums, or the World Association of Zoos and Aquaria; Jiménez-Mena et al., 2016). Moreover, pedigree analysis based on studbooks data is widely recognized as the most important tool to improve management by guiding captive breeding decisions (Nietlisbach et al. 2017; Galla et al. 2020). In the case of Cuvier’s gazelle, whose studbook includes almost 50 years of accurate genealogical information, captive breeding has benefited from this information for guiding genetic management and, therefore, improving its long-term genetic viability. The increasing availability of genetic technology can help overcome pedigrees’ challenges (the expected relatedness between individuals can differ from the realized relatedness, as pedigrees rely on probabilities as opposed to empirical estimates of genome sharing; Galla et al. 2020). However, it is economically unaffordable when the studbooks contain records of over one thousand individuals. A combination of pedigree and molecular information may in fact be the optimal method for measuring genetic relationships, especially when pedigree information is incomplete or shallow, which is not the case of the Cuvier’s gazelle captive breeding programme.

## Supplementary information

Supplementary Material Appendix 1, Appendix 2

## Data Availability

Raw data are available at http://www.eeza.csic.es/documentos/Studbook%202023.pdf.
