# Supplementary material for: Analysing the pedigree to identify undesirable losses of genetic diversity and to prioritize management decisions in captive breeding: a case study
Source: Heredity (Edinb). 2024 Sep 17;133(6):400–9. doi: 10.1038/s41437-024-00723-z (PMC11589844; doi:10.1038/s41437-024-00723-z)
Supplement: Supplementary file 1 — Supplementary Material Appendix 1, Appendix 2 [file 41437_2024_723_MOESM1_ESM.docx]

**SUPPLEMENTARY MATERIAL**

Analysing pedigree to identify undesirable losses

of genetic diversity and to prioritize management decisions

in captive breeding: a case study

**Appendix S1.** Study species

Cuvier’s gazelle, also named Atlas gazelle due to its distribution range in Northern Africa, is medium-sized and sexually dimorphic species, with adult males 24% heavier than adult females (average body mass of adult females: 26 kg; adult males: 34 kg; Moreno and Espeso 2008). Both sexes have horns, being lighter in females. Females are fertile at about 8–9 months and males at 12–13 months. Female reproductive senescence is around 11-12 years. The gestation period is about 5.5 months. Twins represent up to 59% of births (Moreno et al. 2020). It lives in small, familiar groups, which typically contain fewer than eight animals (Kingdon 1997). Harems are quite frequent, with one adult male and a few adult females, accompanied by their recent youngsters (Kingdon 1997).

It is endemic to the mountain and hill ranges of the Maghreb, neighbouring ranges in Morocco, Algeria and Tunisia, where it was historically abundant. Its historical range locally reached the Mediterranean and Atlantic African coast (Cuzin 2003). The whole population has been estimated around 1,750–2,950 individuals (Beudels et al. 2013) with around 900-2,000 individuals in Morocco (IUCN 2018) in a few scattered and largely fragmented populations (see also Aouad et al. 2016 for records of the species in Algeria). This species declined dramatically since the 1950s (Beudels et al. 2005, Beudels et al. 2013, Cuzin et al. 2008), apparently due to excessive hunting, anthropogenic barriers and habitat degradation and only a few small isolated populations seem to remain (IUCN 2018, but see Gil-Sánchez et al. 2017).

Founder individuals of Cuvier’s gazelle captive breeding programme come from Morocco, where its population were homogeneously distributed from southeast to northwest along the Atlas mountains (Cuzin 2003).

**References**

Aouad B, Bounaceur F, Maatoug M (2016) Structure des populations de Gazella cuvieri (Ogilby, 1941) dans la région de Tiaret, Nord-Ouest Algérien. Bull Soc Zool Fr, 141: 141-152

Beudels R., Devillers P, Cuzin F (2013) *Gazella cuvieri* Cuvier's gazelle (Atlas gazelle, Edmi gazelle). In Kingdon J and Hoffmann M, eds., Mammals of Africa, Volume VI, Pigs, Hippopotamus, Chevrotain, Giraffes, Deer and Bovids. Bloomsbury Publishing, London 349-352.

Beudels R., Devillers P, Lafontaine RM, Devillers-Terschuren J, Beudels MO (2005) Sahelo-Saharan Antelopes. Status and Perspectives. CMS Technical Series Publication, No. 11. UNEP/CMS Secretariat, Bonn

Beudels R., Devillers P, Cuzin F (2013) *Gazella cuvieri* Cuvier's gazelle (Atlas gazelle, Edmi gazelle). In Kingdon J and Hoffmann M, eds., Mammals of Africa, Volume VI, Pigs, Hippopotamus, Chevrotain, Giraffes, Deer and Bovids. Bloomsbury Publishing, London 349-352

Cuzin F (2003) Les grands mammiferes du Maroc Meridional (Haut Atlas, Anti Atlas et Sahara): Distribution, écologie et conservation. Thèse de Doctorat. Université Montpellier. Montpellier, France. <https://theses.fr/2003MON2A001>

Cuzin F, Sehhar EA, Wacher T (2008) Strategic Action Plan for the Conservation, Restoration and Management of Ungulates in Morocco (English supplement to Vol.1). Haut Commissariat aux Eaux et Forêts et à la Lutte Contre le Désertification, Projet de Gestions des Aires Protégées, and World Bank Global Environment Facility

Gil-Sánchez JM, Herrera-Sánchez F J, Álvarez B, Arredondo Á, Bautista J, et al (2017) Evaluating methods for surveying the Endangered Cuvier’s gazelle *Gazella cuvieri* in arid landscapes. Oryx 51:648–655

IUCN (2018) Conservation strategy and action plan for Cuvier’s gazelle (*Gazella cuvieri*) in North Africa 2017-2026. IUCN Gland, Switzerland and Malaga, Spain: x + 42 pp. https://doi.org/10.2305/IUCN.CH.2018.02.en

Kingdon J (1997) The Kingdon Field Guide to African Mammals. Academic Press, London and New York

Moreno E, Espeso G (2008) International studbook. Cuvier’s gazelle (Gazella cuvieri). Ayuntamiento de Roquetas de Mar: CSIC, Almería-Madrid

Moreno E, Jebali A, Espeso G, Benzal J (2020) Reintroducing Cuvier’s Gazelle. Better than Expected from Captive-Bred Founders. Global Ecol Conserv 23 (e01094): e01094

**Appendix S2.** Mathematical formulations

Computation of some of the parameters listed in the Materials and Methods sections is detailed below:

- The number of equivalent complete generations traced (*t*; Maignel et al., 1996), was computed as the sum of (1/2)*^n^*, where *n* is the number of generations separating the individual to each known ancestor.
- The effective number of non-founders (*nf_e_*) was, computed following Caballero and Toro (2000), as ${nf_{e}=\left( \frac{1}{f_{g}}-\frac{1}{f_{e}} \right)}^{-1}.$ Note that $\frac{1}{f_{e}}$ is an estimator of the gene diversity or expected heterozygosity in the founder population (GD^*^) while $\frac{1}{f_{g}}$ is an estimator of the gene diversity accounting for unequal contributions of founders (GD). Therefore, $\frac{1}{nf_{e}}$ estimates the genetic diversity lost due to random drift (RD), where RD = GD^*^ - GD.
- Parameters $N_{e}F_{i}$ and $N_{e}C_{ij}$ were computed using individual increase in inbreeding Δ*F*_i_ and coancestry Δ*C*_ij_ following Gutiérrez et al. (2009) and Cervantes et al (2011), respectively, considering $\Delta F_{i}=1-\sqrt[t_{i}-1]{(1-F_{i})}$ and $\Delta C_{ij}=1-\sqrt[{(t}_{i}+t_{j})/2]{(1-C_{ij})},$ where *F*_i_ is the inbreeding coefficient of individual *i*, *C*_ij_ is the coancestry coefficient between individuals *i* and *j*, and *t_i_* and *t_j_* are their respective equivalent complete generations. Finally, effective population sizes were computed using the following formulae: $N_{e}F_{i}=1/(2\bar{\Delta F})$ and $N_{e}C_{ij}=1/(2\bar{\Delta C})$.
- *F_IS_* and *F_ST_* statistics were computed, following Caballero and Toro (2000), from coancestry information as $F_{IS}=\frac{\tilde{F}-\bar{f}}{1-\bar{f}}$, and $F_{ST}=\frac{\bar{f}-\tilde{f}}{1-\tilde{f}}$, where $\tilde{F}$ and $\tilde{f}$are, respectively, the mean inbreeding and the mean coancestry for the entire population, and $\bar{f}$ the average coancestry for each reference population.
- Contributions of geographical populations to total gene diversity were assessed following Caballero and Toro (2002). The average coancestry (Malècot 1948), $\bar{f}$, over the entire population of *N_T_* individuals consisting of *n* subpopulations (each reference population in this study), subpopulation *i* with *N_i_* breeding individuals, is:

$$\bar{f}=\frac{\sum_{i,j=1}^{n} f_{ij}N_{i}N_{j}}{N_{T}^{2}}=\frac{\sum_{i=1}^{n} f_{ii}N_{i}}{N_{T}}-\bar{\mathbf{D}}=\sum_{i=1}^{n} \frac{N_{i}}{N_{T}}\left[f_{ii}-\frac{\sum_{j=1}^{n} \mathbf{D}_{ij}N_{j}}{N_{T}}\right]$$

being *f_ij_* the average pairwise coancestry between individuals of subpopulations *i* and *j*, including all *N_i_ × N_j_* pairs and *f_ii_* the average pairwise coancestry within subpopulation *i* and where ***D_ij_*** is the Nei’s minimum genetic distance (Nei 1987) between subpopulations *i* and *j* computed as ***D_ij_*** = [(*f_ii_* + *f_jj_* )/2] − *f_ij_*. From the formula above it can be noted that $\bar{f}$ is dependent on the within-subpopulation coancestry (first term in the brackets) and the average distance among subpopulations (second term in the brackets). Proportional contribution of each subpopulation to the global coancestry can be computed as the average coancestry of the subpopulation minus its average distance with all the others.

- Following Álvarez et al. (2012), the contribution of the three founder dam lines (defined as an unbroken descent through female animals only from an ancestor to a descendant) was computed from Probability of identity (PI), defined as the probability that two individuals share the same dam line by chance was computed as $\mathrm{PI}=\sum q_{k}^{2}$, where *q_k_* is the frequency of the *k*_th_ dam line. Each *PI* was computed for the founder populations (PI_f_) and each reference populations (PI_r_). Increase in identity was computed as $\Delta\mathrm{PI}=\frac{\mathrm{PI}_{r}-\mathrm{PI}_{f}}{1-\mathrm{PI}_{f}}$. Effective female size was computed as ${mN}_{e}=\frac{1}{\Delta\mathrm{PI}}$.

**References**

Álvarez I, Fernández I, Lorenzo L, Payeras L, Cuervo M, Goyache F (2012) Founder and present maternal diversity in two endangered Spanish horse breeds assessed via pedigree and mitochondrial DNA information. J Anim Breed Genet 129:271-279

Caballero, A, Toro MA (2000) Interrelations between effective population size and other pedigree tools for the management of conserved populations. Genet Res Camb 75:331-343

Caballero, A, Toro MA (2002) Analysis of genetic diversity for the management of conserved subdivided populations. Conserv Genet 3:289–299

Cervantes, I., Goyache, F., Molina, A., Valera, M. & Gutiérrez, J.P. (2011) Estimation of effective population size from the rate of coancestry in pedigreed populations. J Anim Breed Genet 128:56-63

Gutiérrez JP, Cervantes I, Goyache F (2009) Improving the estimation of realized effective population sizes in farm animals. J Anim Breed Genet 126:327-332

Maignel L, Boichard D, Verrier E (1996) Genetic variability of French dairy breeds estimated from pedigree information. Interbull Bull. 14:49-54

Malècot G (1948) Les Mathématiques de l'Hérédite. Masson et Cie, Paris
